# Pembrolizumab combined with stereotactic body radiotherapy in a patient with human immunodeficiency virus and advanced non-small cell lung cancer: a case report

**DOI:** 10.1186/s13256-018-1667-2

**Published:** 2018-04-23

**Authors:** Dongqi Li, Chuanchun He, Yaoxiong Xia, Yaxi Du, Jing Zhang

**Affiliations:** 1 Bone and Soft Tissue Tumors Research Center of Yunnan Province, Department of Orthopaedics, The Third Affiliated Hospital of Kunming Medical University (Tumor Hospital of Yunnan Province), Kunming, Yunnan 650118 People’s Republic of China; 2 Department of Radiotherapy, The Third Affiliated Hospital of Kunming Medical University (Tumor Hospital of Yunnan Province), Kunming, Yunnan 650118 People’s Republic of China; 3 Lung Cancer Research Center, The Third Affiliated Hospital of Kunming Medical University (Tumor Hospital of Yunnan Province), Kunming, Yunnan 650118 People’s Republic of China

**Keywords:** Pembrolizumab, Stereotactic body radiotherapy, Immune-related adverse events, Non-small cell lung cancer, HIV

## Abstract

**Background:**

Pembrolizumab has significantly improved outcomes in patients with advanced non-small cell lung cancer. Combining programmed death-1 inhibitor with stereotactic body radiotherapy showed a slight toxicity and good benefits in recent clinical trials. However, patients infected with human immunodeficiency virus were excluded from most trials because it was assumed that their anti-tumor immunity was compromised compared with immunocompetent patients.

**Case presentation:**

In June 2016, a 52-year-old Chinese man presented with human immunodeficiency virus and lung adenocarcinoma (T1bN3M1b). From November 2016 to December 2016, systemic chemotherapy and palliative radiotherapy for bone metastasis of femoral neck were carried out, but the tumor progressed. In January 2017, after immunochemistry detection of programmed death-1 and programmed death-ligand 1 expression (both > 50%), pembrolizumab was started. Three weeks after pembrolizumab, we combined stereotactic body radiotherapy for the primary lung tumor. He received no comfort and his CD4 lymphocyte count was stable. Human immunodeficiency virus-ribonucleic acid remained below the limits of detection. In March 2017, after three cycles of pembrolizumab and 5 weeks of stereotactic body radiotherapy therapy, he suddenly presented with palpitations. Emergency computed tomography scanning showed massive pericardial effusion and interstitial pneumonia. So we interrupted the pembrolizumab use and initiated treatment with prednisolone 1 mg/kg; however, the tumor progressed. Then, his CD4 lymphocyte count declined. Finally he died in June 2017 due to dyscrasia.

**Conclusions:**

Pembrolizumab combined with SBRT therapy for patients with human immunodeficiency virus infection and non-small cell lung cancer may lead to serious immune-related adverse events and more clinical trials are needed.

## Background

Pembrolizumab, the first Food and Drug Administration (FDA)-approved programmed death-1 (PD-1) inhibitor, has significantly improved outcomes in patients with advanced non-small cell lung cancer (NSCLC) [1].Combining PD-1 inhibitor with stereotactic body radiotherapy (SBRT) showed a slight toxicity and good benefits in recent clinical trials [2, 3]. However, patients with human immunodeficiency virus (HIV) infection were excluded from most trials, because it was assumed that their anti-tumor immunity was compromised compared with immunocompetent patients.

## Case presentation

Here, we report the case of a patient with HIV and advanced NSCLC who was treated with PD-1 inhibitor (pembrolizumab) combined with SBRT.

**Fig. 1:**
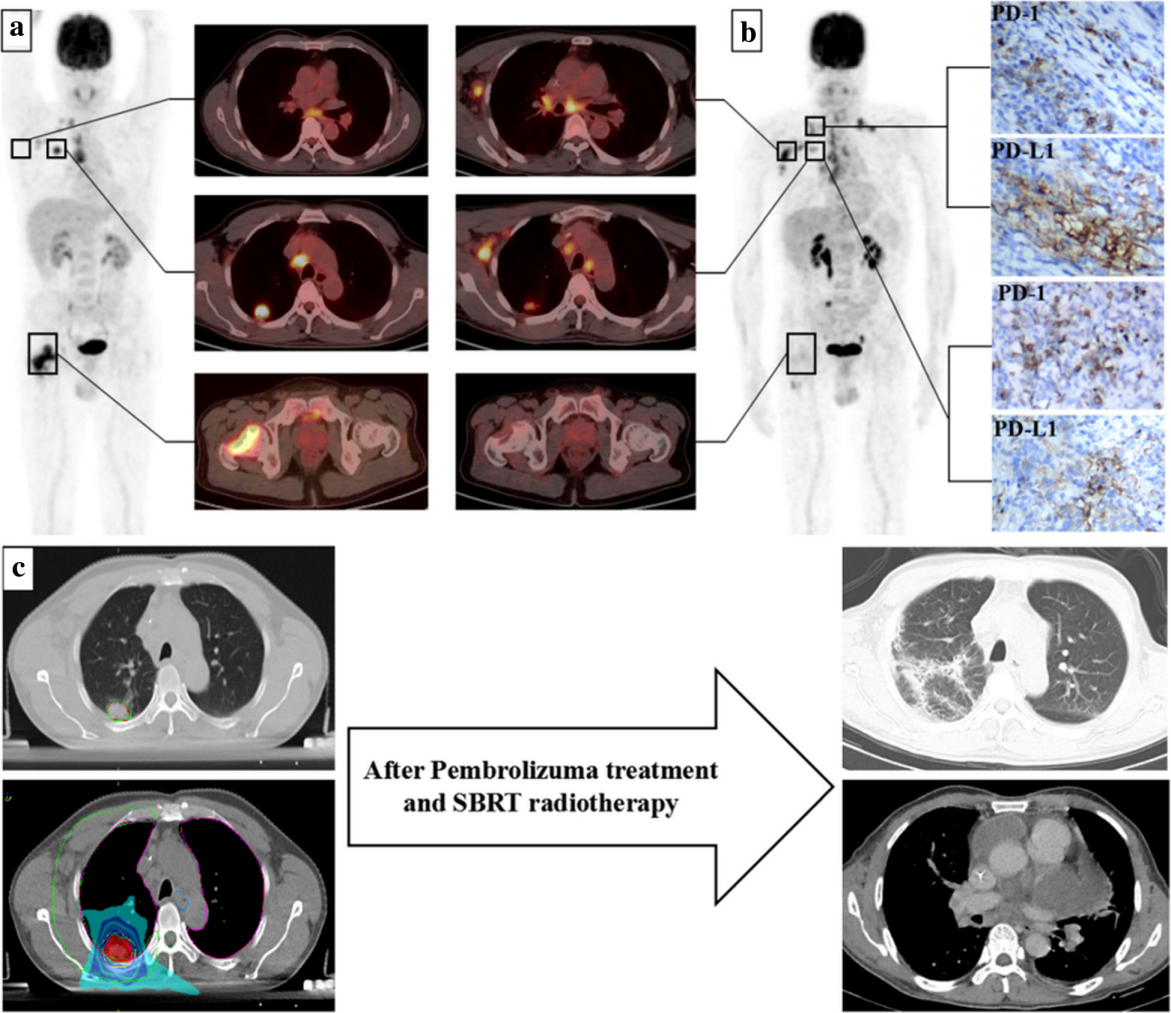
**a** Before treatment, tumors of the lung (posterior segment of upper lobe of right lung) and the ipsilateral femoral neck, as well as a small amount of axillary and cervical lymph nodes, were found by positron emission tomography-computed tomography. **b** After chemoradiotherapy, the tumors in the lung and femoral neck were controlled. However, the tumor of axillary and cervical lymph nodes progressed. Meanwhile, immunochemistry detection showed that programmed death-1 and programmed death-ligand 1 expression were more than 50% in the tumor cells and immune cells of biopsy tissues of the cervical lymph node and primary lung tumor. **c** After three cycles of pembrolizumab and 5 weeks of stereotactic body radiotherapy therapy, the patient presented with pericardial effusion and interstitial pneumonia. *PD-1* programmed death-1, *PD-L1* programmed death-ligand 1, *SBRT* stereotactic body radiotherapy

In June 2016, a 52-year-old Chinese man who had never smoked tobacco, who had an initial diagnosis of HIV infection in 2013 with highly active anti-retroviral therapy, was diagnosed as having advanced lung adenocarcinoma (T1bN3M1b) with *KRAS* mutation (exon 2 deletion). From November 2016 to December 2016, systemic chemotherapy (carboplatin/pemetrexed for two cycles) and palliative radiotherapy for bone metastasis of femoral neck (intensity-modulated radiation therapy, 48 Gy/16 fractions) were carried out, but the tumor progressed with new metastatic lymph nodes (Fig. 1a).

In January 2017, after immunochemistry detection of PD-1 and programmed death-ligand 1 (PD-L1) expression (both > 50%, Fig. 1b), pembrolizumab was started (2 mg/kg, every 3 weeks). Three weeks after pembrolizumab, we combined SBRT for the primary lung tumor (50 Gy/five fractions, every other day). Our patient received no comfort and his CD4 lymphocyte count was stable. Human immunodeficiency virus-ribonucleic acid (HIV-RNA) remained below the limits of detection.

In March 2017, after three cycles of the pembrolizumab and 5 weeks of SBRT therapy, he suddenly presented with palpitations. Emergency computed tomography (CT) scanning showed massive pericardial effusion and interstitial pneumonia (Fig. 1c). So we interrupted the pembrolizumab use and initiated treatment with prednisolone 1 mg/kg; however, the tumor progressed. Then, his CD4 lymphocyte count declined. Finally he died in June 2017 due to dyscrasia.

## Discussion

The use of highly active anti-retroviral therapy has prolonged the survival of patients with HIV, which increased the incidence of HIV-related malignancies, including lung cancer [4]. The treatment of patients with HIV infection and NSCLC has rapidly gained attention. Both mechanisms of pembrolizumab and SBRT therapy aroused anti-tumor effect of immunity cells [5]. To our knowledge, this is the first case report on treating patients with HIV infection and NSCLC by combining pembrolizumab with SBRT consecutively.

The latest two case reports on PD-1 inhibitor in treating patients with HIV infection and NSCLC were inconsistent. Regarding the tumor response, one case had complete remission [6], but the other progressed [7]. Our results were in line with the latter. One reason might be that the anti-tumor effect of immunity cells was weakened after chemoradiotherapy. We also thought that impaired bone marrow reserve resulting from chemoradiotherapy may have contributed to the CD4 cell decline.

It seems that HIV status has no impact on the local tumor immune microenvironment, including immune cell subset (CD3, CD4, CD8, and CD68) infiltration or PD-L1 expression [8]. However, it is difficult to explain the mechanism of rapid immune-related adverse events (IRAEs). The latest retrospective controlled study (*n* = 164) showed that thoracic radiotherapy did not increase the risk of interstitial pneumonia [9]. In this case, we report other IRAEs of pericardial effusion. It is essential to select defining biomarkers that predict immunotherapy response and IRAEs [10].

## Conclusion

Pembrolizumab combined with SBRT therapy for patients with HIV infection and NSCLC may lead to serious IRAEs and more clinical trials are needed.

## Abbreviations

IRAEs: Immune-related adverse events
NSCLC: Non-small cell lung cancer
PD-1: Programmed death-1
PD-L1: Programmed death-ligand 1
SBRT: Stereotactic body radiotherapy
